# Phytocannabinoids in Neurological Diseases: Could They Restore a Physiological GABAergic Transmission?

**DOI:** 10.3390/ijms21030723

**Published:** 2020-01-22

**Authors:** Pierangelo Cifelli, Gabriele Ruffolo, Eleonora De Felice, Veronica Alfano, Erwin Alexander van Vliet, Eleonora Aronica, Eleonora Palma

**Affiliations:** 1 IRCCS Neuromed, Pozzilli, 86077 Isernia, Italy; 2 IRCCS San Raffaele Pisana, 00163 Rome, Italy; gabriele.ruffolo@uniroma1.it (G.R.); eleonora.df.22@gmail.com (E.D.F.); 3 Department of Physiology and Pharmacology, laboratory affiliated to Istituto Pasteur Italia, University of Rome Sapienza, 00185 Rome, Italy; veronica.alfano@uniroma1.it; 4 Amsterdam UMC, University of Amsterdam, Department of (Neuro) Pathology, 1105 Amsterdam, The Netherlands; e.a.vanVliet@uva.nl (E.A.v.V.); e.aronica@amsterdamumc.nl (E.A.); 5 Swammerdam Institute for Life Sciences, Center for Neuroscience, University of Amsterdam, 1090 Amsterdam, The Netherlands; 6 Stichting Epilepsie Instellingen Nederland (SEIN), 0397 Heemstede, The Netherlands

**Keywords:** GABA_A_Rs, cannabinoids, neurotransmission, endocannabinoid system

## Abstract

γ-Aminobutyric acid type A receptors (GABA_A_Rs) are the main inhibitory mediators in the central nervous system (CNS). GABA_A_Rs are pentameric ligand gated ion channels, and the main subunit composition is usually 2α2βγ, with various isotypes assembled within a set of 19 different subunits. The inhibitory function is mediated by chloride ion movement across the GABA_A_Rs, activated by synaptic GABA release, reducing neuronal excitability in the adult CNS. Several studies highlighted the importance of GABA-mediated transmission during neuro-development, and its involvement in different neurological and neurodevelopmental diseases, from anxiety to epilepsy. However, while it is well known how different classes of drugs are able to modulate the GABA_A_Rs function (benzodiazepines, barbiturates, neurosteroids, alcohol), up to now little is known about GABA_A_Rs and cannabinoids interaction in the CNS. Endocannabinoids and phytocannabinoids are lately emerging as a new class of promising drugs for a wide range of neurological conditions, but their safety as medication, and their mechanisms of action are still to be fully elucidated. In this review, we will focus our attention on two of the most promising molecules (Δ9-tetrahydrocannabinol; Δ9-THC and cannabidiol; CBD) of this new class of drugs and their possible mechanism of action on GABA_A_Rs.

## 1. Introduction

GABA is the main inhibitory neurotransmitter in the central nervous system (CNS), able to bind three different classes of γ-Aminobutyric acid type A receptors (GABARs): GABA_A_Rs, GABA_B_Rs, and GABA_C_Rs. In this review we will focus our attention on GABA_A_Rs, and their possible interaction with a new class of drugs: phytocannabinoids (pCBs). GABA_A_Rs are chloride-permeable ligand-gated ion channels (LGIC), which induce a neuronal hyperpolarization and reduce neuronal excitability in the CNS [1,2]. GABA_A_R impairment seems to be involved in the pathogenesis of several neurodevelopmental diseases, epileptic syndromes, and cognitive dysfunctions [3], conditions that can often coexist. Indeed, both benzodiazepines (BDZ) and barbiturates (BBT) specifically target GABA_A_Rs and are used in the treatment of anxiety, sleep disorders, and seizures, especially during status epilepticus. However, these classical GABAergic agents often face problems linked to tolerability and strong side-effects. To address this important issue, new compounds are currently under investigation. Among these latter, *Cannabis sativa* (*Cannabis s.*) and its derivatives pCBs have had more and more attention within the scientific community in the past years [4,5], because of their vast therapeutic potential possibly involving GABAergic modulation.

*Cannabis s.* is a plant that contains more than 500 components, among which 104 cannabinoids have been presently identified [6]. However, two of these compounds have been deeply investigated in the last years: Δ9-tetrahydrocannabinol (Δ9-THC) and cannabidiol (CBD).

It is also important to keep in mind the presence of endogenous cannabinoids molecules, as 2-Arachidonoylglycerol (2-AG) and anandamide (AEA). These class of molecules, also called endocannabinoids (ECs), are able to interact with G protein-coupled receptors (GPCRs), the cannabinoid CB1 and CB2 receptors (CB1R and CB2R) [7]. The ECs and the CB receptors are part of a complex signaling system deeply involved in mammalian physiology and pathology, and for this reason represents an interesting target for the developing of new therapeutic compounds [7]. AEA preferentially bind and activates CB1R, while 2-AG can activate both CB1R and CB2R [8].

Similarly, Δ9-THC has the capacity to bind both the ECs receptors CB1 and CB2 (CBRs), and has strong psychoactive effects, while CBD’s target and its own mechanism of action seem to be slightly shader. Indeed, CBD is a non-psychoactive compound with a better safety profile if compared with Δ9-THC: it seems to be well tolerated at high doses in both animal models and humans, it does not alter heart rate, blood pressure, or body temperature and has no effects on locomotor activity or superior cognitive functions [9]. This good safety profile is probably related to its pharmacodynamics, since it binds with low affinity both to CB1Rs and CB2Rs [10,11,12].

This last detail, though, paved the way for an intensive investigation of the possible alternative targets of this compound but, at the current state of the art, none of the proposed mechanisms seem to fully explain its clinical efficacy [13].

## 2. Pharmacological Targets for Major pCBs

Δ9-THC and CBD are two of the most studied molecules among pCBs [14]; while on one hand they share common chemical structures, they profoundly differ in terms of pharmacodynamic properties. Indeed, while Δ9-THC can bind and activate the two most widespread population of metabotropic receptors in the human brain [15], namely CB1R and CB2R and has strong psychoactive properties, CBD action seems not to largely depend on the activation of these receptors and its own mechanism of action is still poorly understood. Furthermore, and most importantly, psychoactive effects have not been reported for this compound. As mentioned above, CBD is able to bind CBRs only at high concentrations [16] and surprisingly it can act on CB2Rs as an inverse agonist [17], thus reducing its activity.

It is also important to keep in mind the immune-system regulatory effects of both ECs and pCBs. Indeed, several papers highlighted how these compounds can modulate different subpopulations of immune cells. In detail, it was shown that Δ9-THC is able to induce suppression of T-cell immune-response by means of selective apoptosis and to significantly reduce inflammatory cytokines and chemokines release, such as IL-1α, IL-1β, IL-6, and TNF-α in different in vitro models [18]. Moreover, it was also shown the capacity of both ECs and pCBs to modulate immune response on astrocytes by acting mostly on CB1Rs [19]. Notably, it was also reported CBD’s capacity to modulate immune-system response. CBD is able, as shown for Δ9-THC, to strongly inhibit IL-6 release in different preclinical models of inflammatory diseases, such as diabetes, asthma, pancreatitis, and hepatitis [20]. However, since the molecular targets of these compounds are numerous and the overall effect on the immune system is still partially understood, there are still doubts about their use for human inflammatory diseases [21,22] and further investigations are needed in order to better clarify the pCBs effects on immune system function. For a quick review of the overall targets described below refer to Table 1.

### 2.1. Transient Receptor Potential Vanilloid Sub-Family

The first scientific evidence that CBD can bind and modulate other receptors than CB1Rs and CB2Rs was published in 2001 by Bisogno et al. [23]. These authors demonstrated that CBD binds “transient receptor potential vanilloid type 1” (TRPV1) and interacts with proteins involved in the inactivation of the endogenous cannabinoid, anandamide (AEA). These families of proteins seem to be involved in several neurodegenerative conditions and may represent an important target to modulate with new pharmacological compounds. TRPV proteins contribute to heat perception and inflammatory reactions and mediate pain sensation. CBD acts on TRPV channels as an agonist, especially on TRPV1 channel sub-type, inducing activation, dephosphorylation, and strong desensitization, which in turn decreases intracellular calcium levels and neuronal excitability [36], thus accounting for both the CBD anti-nociceptive and anti-convulsant effects.

### 2.2. Opioid Receptors

It was also reported that CBD and THC act as an allosteric modulator at μ and δ opioid receptors. Indeed, Kathmann and colleagues, using rat cerebral cortex membrane homogenates, performed kinetic binding studies in order to evaluate the allosteric interactions on this class of G protein-coupled receptors. This study demonstrated, for the first time, the capability of these compounds to strongly modulate the μ and δ opioid receptors adding more evidence to explain pCBs anti-nociceptive effects [36].

### 2.3. G Protein-Coupled Receptors GPR55

The G protein-coupled receptor GPR55 acts as a “gate” regulating glutamate release with a calcium-dependent mechanism. CBD seems to antagonize the GPR55 activation, thus reducing glutamate release and neuro-excitability in the CNS [25,37], which partially explains pCBs’ anti-convulsive action on epileptic pharmaco-resistant patients [12].

### 2.4. Voltage Gated Calcium Channels 

Another interesting pharmacological target for pCBs is represented by the family of voltage-gated calcium channels (VGCC), deeply involved in the regulation of excitability on CNS [38]. Indeed, both Δ9-THC and CBD are able to strongly modulate this class of receptors. In particular, by acting on transient-type (T-type) and long-lasting-type (L-type) VGCC, these compounds could affect pain modulation/transmission and overall CNS excitability, thus partially explaining the antinociceptive and anti-convulsant effect showed by pCBs [26,38].

### 2.5. Glycine Receptors

Another target proposed for pCBs is represented by Glycine receptors (GlyRs). This family of pentameric ionotropic receptors are chloride permeable and are strongly involved in neuropathic pain and inflammation [27,39]. High concentrations of CBD (>100 μM) are able to directly activate α1 containing GlyRs, even if the physiological significance of this effect remains still uncertain. However, it was also demonstrated that CBD can modulate α3 containing GlyRs at lower concentrations (1 μM), indicating that this effect is strongly related to GlyRs stoichiometry. Further studies are required to better understand the action of pCBs on GlyRs and its physiological role in neurotransmission.

### 2.6. Serotonin Receptors

This class of receptors (5-HTRs) is strongly involved in stress-related disorders and in the cardiocirculatory function. It was demonstrated that CBD can act as direct agonist on 5-HT1ARs and as partial agonist on 5-HT2ARs [28]. The pharmacological interaction between CBD and 5-HTRs was then confirmed in other in vivo studies, where activation of 5-HTRs by CBD was able to reduce the stress response in male rats, indicating significant panicolytic effects [29,39].

### 2.7. Acetylcholine Receptors

There is also evidence that CBD can modulate another important class of receptors, represented by nicotinic acetylcholine receptors (nAChRs). Indeed, CBD can inhibit α-7-nAChR in a dose-dependent manner and induces a reduction of ACh-evoked currents amplitude in rat hippocampal slices [31]. This evidence needs to be further investigated in preclinical models of disease in order to understand the physiological meaning.

### 2.8. Voltage Gated Sodium Channels 

CBD is able to block voltage gated sodium channels (VGSC) acting as an antagonist at micromolar concentration [32]. In detail, these studies were performed using different in vitro models as rat brain slices, cultured mouse cortical neurons, and human SH-SY5Y cell culture. CBD was tested (1–10 μM) on two different types of VGSCs: respectively, the Nav1.1 and Nav1.2 subtype. Results from these tests highlighted the capacity of CBD to inhibit these channels, without a clear dose-response relationship. Interestingly, also another minor cannabinoid, cannabidivarine (CBDV) was able to block this population of ion channels. However, it is important to notice that when these molecules were used in animal models of epilepsy, the blockade of VGSCs was not able to confer anti-convulsant effects alone, thus indicating that other mechanisms are involved in anti-seizure efficacy of pCBs [40].

## 3. GABA_A_ Receptors

GABA_A_Rs are members of the rapid-acting, LGIC receptor category. The composition of these receptors arises from 19 different subunits (α1–6, β1–3, γ1–3, δ ε θ π, and 1–3). Usually they are formed by two copies of an α subunit, two copies of a β subunit, and one copy of either a γ subunit, or another such as δ. The main composition of GABA_A_Rs is related to different factors, such as age, the brain area considered, and the cellular population. Indeed, the pharmacological properties of this chloride ion channel strictly depend on receptor subunit composition [41] and arrangement [42]. If this high variability in subunit composition makes it difficult to understand their specific actions, on the other hand it may help to develop selective compounds able to bind and modulate specific GABA_A_Rs in different brain areas. Moreover, GABA_A_Rs may be located on synapses or extrasynaptically; synaptic GABA_A_Rs mediate phasic inhibition, whereas extrasynaptic GABA_A_Rs receptors mediate tonic inhibition [43,44]. In detail, extrasynaptic GABA_A_Rs are usually formed by α4, α5, and α6 subunits, with the presence of the δ-subunit [43,44].

It was also described that extrasynaptic and synaptic GABA_A_Rs subunit composition and localization is modulated by the neuroendocrine system, thus adding another complication in the knowledge of GABA_A_Rs functions [45].

GABA_A_Rs are also present on peripheral blood mononuclear cells (PBMC) such as T cells, B cells, and NK cells. In particular, by using RT-PCR the presence of α1, α3, β2, β3, δ, and ɛ subunits was demonstrated. Application of GABA was able to activate these receptors, and bicuculline antagonized this effect, indicating thus that human PBMC express functional GABA_A_Rs and that these receptors may be involved in the modulation of immune response, [46] especially in neurological diseases characterized by neuro-inflammatory processes.

This sub-family of GABA_A_Rs seems to be implicated in several neurological diseases [47] and may affect seizure susceptibility [48] thus making them an interesting target for new pharmacological compounds. Indeed, GABA_A_Rs dysfunction is involved in epileptic disorders [49], drug addiction [50], Huntington disease [51], chronic stress, and anxiety [52]. More specifically, GABA_A_Rs undergo structural and functional alterations due to a significant change in subunit composition both in animal models of temporal lobe epilepsy (TLE) [53] and in pharmaco-resistant TLE patients, partially explaining the increased cerebral network excitability [54]. Indeed, a significant shift between the α1 and the α4/α5 subunits has been reported in epileptic patients compared with non-epileptic controls, especially in the hippocampal formation. This may partially explain the increased excitability of this area in epileptic conditions [54].

Another interesting alteration, typically associated with pharmaco-resistant epilepsy, is the increased tendency of GABA_A_Rs to desensitize during massive GABA release at synaptic level. This phenomenon is characterized by an anomalous response with reduced GABA evoked currents during sustained GABA activation, making the whole neuronal network more prone to developing seizures [55]. This phenomenon, called GABA_A_Rs “run-down” has been shown in both animal models of epilepsy [53,56] and in human tissues from epileptic patients [55,57]. GABA_A_Rs “run-down” seems to be related to altered intracellular phosphorylation processes and represents an interesting target to “stabilize” GABA_A_Rs and thus to reduce hyperexcitation of the whole nervous system [58]. Moreover, GABA_A_Rs “run-down” can be also targeted by several compounds, from neurotrophic factors such as the brain derived neurotrophic factor (BDNF), [56] and fractalkine/CX3CL1 [59] to anti-epileptic drugs (AEDs), as lacosamide and levetiracetam [34].

### Cannabinoids and GABA_A_Rs

The first evidence that cannabinoids could modulate GABA_A_Rs came from Sigel and colleagues [60]. They described, for the first time, how the endocannabinoid 2-AG was able to increase GABA_A_ currents elicited by GABA application in a dose-dependent manner. In this paper the authors also highlighted that the 2-AG mediated effect requires the presence of the GABA_A_Rs β subunits.

Starting from this milestone paper, several authors described how not only ECs are able to modulate and potentiate GABA_A_ mediated currents, but also pCBs. The first demonstration that CBD acts at GABA_A_Rs level [33] was highlighted by using recombinant DNA injection into *Xenopus laevis* oocytes. In this very elegant paper, the authors were able to demonstrate that CBD could increase GABA_A_ mediated currents in a dose dependent manner, demonstrating that β subunits represent the main binding site for both endocannabinoids and CBD on GABA_A_Rs. Importantly, it was also demonstrated that, Δ(9)-THC does not act on GABA_A_Rs [61], thus indicating the major role of CBD as GABA_A_Rs modulator.

Other studies also investigated the involvement of CBD on GABA_A_Rs mediated transmission: by using human tissues obtained from patients suffering of Dravet syndrome (DS) and tuberous sclerosis complex (TSC) it was demonstrated that CBD at 5 μM concentration is able to significantly increase GABA-evoked currents amplitude, acting as a positive allosteric modulator. This effect was totally reverted after a short wash-out [62] and was comparable to that of a typical benzodiazepine (BDZ), i.e., flunitrazepam. Indeed, CBD was able to significantly increase evoked GABA_A_Rs currents, both on α1- and α2-containing receptors, with an efficacy that was very similar to the flunitrazepam. Interestingly, CBD’s effect still persisted on γ less receptors, as opposed to flunitrazepam which totally lost its efficacy, thus suggesting that CBD and BDZ act on different binding sites. In another study, the pCB CBDV was also tested on GABAergic transmission. CBDV is a promising molecule present in the *Cannabis s.* plant because it represents a CBD propyl-analogue without any psychoactive effect. This compound was able to significantly reduce both severity and duration of seizures in a broad range of preclinical models of epilepsy [63]. Later, it was also demonstrated that the CBDV anti-convulsant effect was CBRs independent [64], while its effects have been related to a TRPV mediated mechanism. However, these results do not fully explain the CBDV strong anti-convulsant efficacy shown in the preclinical models of epilepsy. Notably, in a recent paper [35] it has been shown that CBDV could act, as for CBD, on GABA_A_Rs modulating its function. In fact, CBDV was able to significantly recover the GABAergic “run-down” phenomenon [65] in a TRPV-independent manner, probably linked to intracellular processes of phosphorylation/dephosphorylation on GABA_A_Rs [35].

## 4. Cannabinoids on Neurological Diseases with GABA Involvement

**Figure 1:**
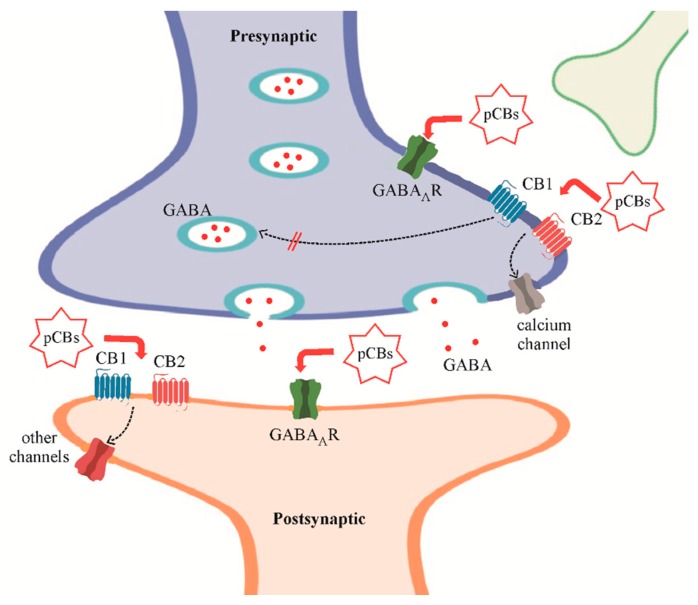
The major phytocannabinoids tetrahydrocannabinol (THC), cannabidiol (CBD), and cannabidivarine (CBDV) (phytocannabinoids—pCBs) can modulate γ-Aminobutyric acid type A receptors (GABA_A_Rs) both directly [33,61] and through the activation of cannabinoid receptors 1 (CB1) and 2 (CB2) [66]). As shown, pCBs targets are located both at the presynaptic and postsynaptic terminals. In detail, pCBs can modulate GABA release by acting on presynaptic CBRs and enhance the postsynaptic GABAergic activity by potentiating GABA_A_R currents.

The neuro-protective effect of pCBs on the CNS is one of the key points that could justify the clinical use of these compounds in neurological disorders. However, while this effect could be explained easily by Δ9-THC action on CB1Rs [66], CBD’s mechanism of action in CNS diseases is still partially unclear. Here, we briefly describe the latest data about the rationale for using pCBs in different pathological conditions, especially underlying how the CBD could affect the altered GABARs function in CNS (Figure 1).

### 4.1. Parkinson Disease (PD) and Motor Functions Impairment

The first evidence for a role of the endocannabiod system (ECS) and pCBs on PD came out around 20 years ago, when, using an animal model of PD, it has been shown a reduction of dyskinesia/akinesia-related symptoms stimulating CBRs with the non-selective agonist for both CB1Rs and CB2Rs, WIN-55,212-2. However, authors did not notice any improvement on other parkinsonian symptoms (tremors, rigidity) suggesting that the effect on akinesia could be related to a pCBs-mediated activation of a pathway linking the striatum indirectly to basal ganglia outputs via the lateral globus pallidus and the subthalamic nucleus [67,68].

Interestingly, in a small pilot study with patients, the improvement of dyskinesia was fully confirmed [69] highlighting a novel GABA-related mechanism of pCBs action. Indeed, the stimulation of CBRs localized in the globus pallidus nucleus reduced the synaptic GABA re-uptake enhancing the GABAergic transmission in the circuit [69]. However, despite of the large number of studies performed on preclinical animal models of PD, clinical trials results using pCBs and ECs, are still not conclusive and often controversial, thus indicating the need of more accurate studies on large PD patient cohorts [70].

### 4.2. Alzheimer Disease (AD)

Concerning the role of ECs and pCBs on neurological conditions characterized by loss of memory and dementia-like phenotype, several preclinical studies were published highlighting a possible use for these compounds in treating these pathologies. GABA_A_Rs seem to have an important role in this neurodegenerative disorder, especially for impairment of memory processes. Indeed, in both human and preclinical models of disease a selective loss of two populations of GABAergic interneurons has been described, namely somatostatin and parvalbumin positive interneurons in the perirhinal cortex [71]. This increased loss of inhibitory elements may thus explain the altered brain rhythm of these subjects often accompanied with the cognitive impairment. In another study performed using AβPP/PS1 transgenic mice model of AD, it has been also demonstrated that both Δ9-THC and CBD were able to significantly recover the memory deficit, even at late stage of disease by modulating both glutamatergic and GABAergic transmission [72]. Interestingly, in two recent studies made on a large cohort of patients (Babiloni et al., 2019a, Babiloni et al. 2019b, unpublished data) affected by mild cognitive impairment (MCI) with or without diagnosis of AD, it has been demonstrated the presence, by means of electroencephalographic (EEG) recordings, of epileptic-like activity without any sign of seizures phenotype. The use of pCBs could recover the cognitive impairment of these patients, removing aberrant activity from CNS circuits involved in memory functions. AD is also characterized by significant alterations of social interactions and facial recognition; using a transgenic model of AD it was demonstrated the CBD’s ability to prevent the development of this social recognition impairment [73].

### 4.3. Amyotrophic Lateral Sclerosis (ALS)

ALS also known as Lou Gehrig’s disease, is a motoneuronal degenerative disease with a poor prognosis and without an effective therapy that unavoidably leads to patients’ death [74]. ALS is characterized by an extensive loss of motoneurons in the cerebrospinal axis, except for those motoneurons that control eye movements and bladder contraction. ALS is clinically characterized by stiff muscles, muscle twitching, and gradually worsening weakness due to muscle atrophy [75]. While the exact pathophysiological mechanisms are still far from being completely elucidated, data supporting a GABA_A_RS involvement were partially clarified. Indeed, it was demonstrated a strong and linear correlation between the vulnerability of a different subpopulation of motoneurons and the subunit composition of GABA_A_RS on these cells. Accordingly, a different α-subunit rearrangement on ALS-vulnerable motoneurons has been found when compared with ALS-resistant motoneurons [75]. Moreover, using cultured cortical neurons from a genetic model of ALS (G93A), it has been confirmed a GABAergic impairment characterized by an evident GABA_A_Rs stoichiometric rearrangement [76]. While the use of pCBs to treat ALS patients is still far from being approved, preliminary data coming from animal models showed possible beneficial effects, even if further studies are needed. Rossi et al., using a G93A-SOD1 ALS mouse model showed how the pharmacological stimulation of both CB1 and CB2Rs was able to significantly reduce glutamatergic and GABAergic neurotransmission, and how these receptors were overexpressed in the ALS mice when compared with control animals [77].

### 4.4. Autism Spectrum Disorders (ASD)

GABA and ECs could have a relevant role in the neurodevelopmental alterations leading to ASD. Indeed, neurodevelopmental impairment of GABAergic transmission seems to be constantly present in autism-related disorders [78,79].

On the other hand, the involvement of ECs has been investigated already in many animal models with autism-like behavior (like Fmr1 mice, NLGN3 and BTBR mice) and, in most of the cases, a dysregulation of CB1 and CB2Rs has been indicated as possible culprit of the cognitive deficits [80].

Furthermore, a recent study pointed out that children afflicted by ASD have significantly reduced circulating levels of the main ECs [81], suggesting an important role of these compounds in the early stages of the disease.

The importance of cannabinoids in ASD is not limited to endogenous mediators. Indeed, administration of pCBs has been proven very useful in the treatment of epileptic seizures and social deficits in a murine model of Dravet syndrome (C57BL/6J DS mice), a condition invariably associated with ASD, and this beneficial effect could be mediated by the enhancement of inhibitory neuronal signaling [82].

On the wave of these encouraging findings, pCBs could be possible candidates for the treatment of ASD patients. However, because of the residual uncertainty concerning pCBs’ multiple mechanisms of action, there is not yet a unanimous consensus on this issue [83].

### 4.5. Epilepsy

The first papers describing anti-convulsant effect of *Cannabis s.* came from the early 1970s [83,84,85]. These studies highlighted the role for Δ9-THC for this effect, even if the precise mechanism of action was not clear [86,87]. However, up to now, the main actor as an anti-convulsant agent is CBD. Indeed, from June 2018 the US Food and Drugs Administration approved the use of CBD drug to treat two rare and strongly drug resistant epileptic syndromes: Dravet syndrome (DS) and Lennox-Gastaut syndrome (LGS) [88]. Later, the same was also approved by European Medicine Agency [89].

However, since in the clinical trials CBD was often administered in combination with clobazam (CBZ) indicating that some pharmacokinetic interactions could not be excluded, some doubts about the real effectiveness of CBD had risen [90].

Since CBD acts as positive allosteric modulator on GABA_A_Rs [33,61], the same pharmacological target of CBZ, Anderson and colleagues investigated, by using a murine model of DS, the possible interaction between the two compounds. CBD strongly inhibited CYP3A4 and CYP2C19, responsible for clobazam metabolism in the liver, thus increasing plasma concentrations of both CBZ and its active metabolite N-CBZ and prolonging their half-lives [91]. The co-application of both compounds significantly increased the anti-convulsant efficacy versus the single application, increasing also the lifespan of the genetic mice model.

Another controversial point regards the adverse effects (AE) of CBD and its behavior on long-term administration. Recently, a review highlighted, through qualitative and meta-analytic data analysis, the efficacy of CBD and *cannabis* derivates in reducing both seizure frequency and severity, taking in account the AE in the treated patients [92]. From these data it was revealed that that CBD was significantly effective at reducing both seizure frequency and severity in pharmaco-resistant epileptic patients, while the AE were rare and often temporary. Indeed, the most common AE reported were in the order: drowsiness, diarrhea, and pyrexia, and all of them were reversible when CBD therapy was withdrawn [92]. Despite its approval for only two forms of pharmaco-resistant epileptic conditions, preclinical studies were conducted also for other epileptic syndromes. Gu and colleagues, using a mouse model of Angelman syndrome, a neurodevelopmental disorder characterized by cognitive impairment, lack of speech, ataxia, EEG abnormalities, and pharmaco-resistant epilepsy, were able to significantly reduce hyperthermia- and acoustically induced seizures, with a reduction of epileptic activity in EEG recordings. Furthermore, CBD application strongly reduced the theta and delta EEG rhythms that are typical in this condition, without any sign of motor activity impairment [93]. However, further studies are needed to better explain the effect of CBD administration in these pathological conditions.

## 5. Discussion

In the last years pCBs are emerging as promising compounds to treat several pathological conditions, both central and peripheral, and for this reason, lately, the number of published papers regarding this topic has been significantly increasing. However, while the use of pCBs, especially CBD, was approved to treat two pharmaco-resistant epileptic syndromes, such as DS and LGS, their use for other conditions still presents some concerns. In this review, we present and discuss the latest published data regarding the GABA_A_Rs modulation by pCBs, in order to better understand how other pathological conditions could be treated with these compounds. While the action on presynaptic glutamatergic and GABAergic transmission is well known and dependent on CBRs activation, the binding of pCBs on non-CBRs putative targets, need to be taken into account to fully understand the potential therapeutic efficacy of pCBs. In particular, the pCBs action on GABA_A_Rs could open the way to the development of new therapeutic approaches for different pathological conditions. Indeed, GABA_A_Rs impairment is deeply involved in neurodevelopmental diseases, from intellectual disability and ASD to pharmaco-resistant epileptic syndromes, such as fragile X syndrome, Rett syndrome, and DS [94]. Moreover, neurodegenerative diseases have lately emerged as conditions showing GABAergic transmission alterations and GABA_A_Rs functional impairment, thus suggesting this family of receptors as a useful pharmacological target to treat these conditions. It is important to notice that for most of these neurodegenerative diseases, the pharmacological treatment is only symptomatic, without a real disease-modifying effect. The GABA_A_Rs modulation, as highlighted by preclinical studies could thus represent a target not only to treat the symptoms of the diseases but also to delay the onset of their clinical features [94]. However, while at preclinical level the pCBs mechanisms of action are becoming clearer, data from double blind multicentric clinical trials is still lacking and, as a consequence, it is still very difficult to clearly state which conditions could benefit from pCBs treatment.

Moreover, in the last decades, medicinal-grade *cannabis* has been legalized in various countries, but often the high costs of pharmaceutical grade compounds lead patients to use products purchased in shops which do not guarantee standardized production protocols. Indeed, quality, purity, and concentrations of pCBs may profoundly differ [95], and most importantly they are often used without proper medical supervision [96]. This last point is particularly relevant due to the potential addiction that, even if lower compared to other drugs, could still be present in a small percentage of patients under *cannabis* therapy, as shown by Zehra and colleagues in their recent review [97]. Further studies and randomized clinical trials focused on using pure compounds and insight regarding the mechanisms of action of CBs are urgently needed in order to better understand their safety profile on long term use, and their real efficacy in treating pathological conditions involving altered GABAergic neurotransmission in CNS diseases.

## Figures and Tables

**Table 1:** Summary of main targets of pCBs.

Target	pCBs Tested	Concentration	Experimental Model	References
Vanilloid receptor type 1	CBD	10 μM	human hembryonic kidney cells (HEK)	Bisogno et al, 2001 [23]
Opioid receptors	THC and CBD	30 μM for both compounds	rat cerebral cortex membrane homogenates	Kathmann et al, 2006 [24]
G protein-coupled receptor GPR55	THC and CBD	from 500 nM to 2.5 μM	human embryonic kidney (HEK293s) cells	Ryberg et al, 2007 [25]
voltage-gated calcium channels	THC and CBD	from 1 μM to 30 μM	human embryonic kidney (HEK293s) cells	Ross et al, 2007 [26]
Glycine receptors	CBD	from 1 μM to 100 μM	in vivo mice model of chronic pain	Xiong et al, 2012 [27]
Serotonin receptors	(1) CBD (2) CBD (3) CBD	(1) 16 μM (2) from 1 to 20 mg/Kg (3) from 30 to 60 nmol	(1) Chinese Hamster Ovary (CHO) cells (2) in vivo rat model of restrain (3) in vivo rat model of pain	(1) Russo et al, 2005 [28] (2) Resstel et al, 2007 [29] (3) Soares et al, 2010 [30]
Acetylcholine receptors	CBD	10 μM	Xenopus oocytes	Mahgoub et al, 2010 [31]
Voltage gated sodium channels	THC and CBD	from 1 to 10 μM	human embryonic kidney cells (HEK) and human iPSC neurons	Ghovanloo et al, 2018 [32]
GABA_A_ receptors	(1) THC and CBD (2) CBD (3) CBDV	(1) from 0.1 to 100 μM (2) 5 μM (3) 1 μM	(1) human cDNA in Xenopus oocytes (2) surgical human Dravet cortical tissue in Xenopus oocytes (3) surgical cortical human epileptic tissue	(1) Bakas et al, 2017 [33] (2) Ruffolo et al, 2018 [34] (3) Morano et al, 2016 [35]
